# Need for cognition does not account for individual differences in metacontrol of decision making

**DOI:** 10.1038/s41598-022-12341-y

**Published:** 2022-05-17

**Authors:** Florian Bolenz, Maxine F. Profitt, Fabian Stechbarth, Ben Eppinger, Alexander Strobel

**Affiliations:** 1 Faculty of Psychology, Technische Universität Dresden, Dresden, Germany; 2 Max Planck Institute for Human Development, Lentzeallee 94, 14195 Berlin, Germany; 3 Cluster of Excellence “Science of Intelligence”, Technische Universität Berlin, Berlin, Germany; 4 Department of Psychology, Concordia University, Montreal, Canada; 5 PERFORM Centre, Concordia University, Montreal, Canada

**Keywords:** Psychology, Human behaviour

## Abstract

Humans show metacontrol of decision making, that is they adapt their reliance on decision-making strategies toward situational differences such as differences in reward magnitude. Specifically, when higher rewards are at stake, individuals increase reliance on a more accurate but cognitively effortful strategy. We investigated whether the personality trait Need for Cognition (NFC) explains individual differences in metacontrol. Based on findings of cognitive effort expenditure in executive functions, we expected more metacontrol in individuals low in NFC. In two independent studies, metacontrol was assessed by means of a decision-making task that dissociates different reinforcement-learning strategies and in which reward magnitude was manipulated across trials. In contrast to our expectations, NFC did not account for individual differences in metacontrol of decision making. In fact, a Bayesian analysis provided moderate to strong evidence against a relationship between NFC and metacontrol. Beyond this, there was no consistent evidence for relationship between NFC and overall model-based decision making. These findings show that the effect of rewards on the engagement of effortful decision-making strategies is largely independent of the intrinsic motivation for engaging in cognitively effortful tasks and suggest a differential role of NFC for the regulation of cognitive effort in decision making and executive functions.

Human decision making can be guided by different strategies^1^. The framework of Reinforcement Learning^2,3^ discriminates between at least two strategies. First, a *model-free strategy* guides decisions based on previously learnt action-reward associations. This strategy is considered to be computationally simple but can be inaccurate in dynamically changing environments. Second, a *model-based strategy* guides decisions by using a mental model of the environment to predict the consequences of potential decisions. This strategy is usually more accurate, but also requires more cognitive effort because it relies at least partly on executive functions^4,5^.

The regulation of cognitive processes relying on executive functions is called *metacontrol*^6^ and humans show metacontrol of decision-making strategies, that is they adapt their relative reliance on a model-based strategy (vs. a model-free strategy) to situational demands^7,8^. For example, people shift more toward a model-based strategy when rewards become higher and thus investing into the more effortful strategy also pays off more^7^. Following the definition of metacontrol as the *regulation* of decision-making strategies, pronounced metacontrol should thus be reflected in a strong upregulation or downregulation of a strategy when the situational demands change. Here, we will focus on metacontrol in response to changes in reward magnitude which is most commonly studied in studies investigating individual differences in metacontrol of decision-making strategies^9–11^.

Previous work has emphasized the reliance on a model-based strategy as an important computational phenotype providing mechanistic explanations for behavioral and psychological differences between individuals^12^. For example, reduced model-based control has been linked to psychopathological traits^11,13^ suggesting a role as a transdiagnostic impairment^14^. It is an open question whether there are also complementary groups of individuals on the other side of this spectrum showing inflexibly high model-based control. To address this question, we investigated the personality trait Need for Cognition (NFC)^15^ as a potential explanation for reduced, context-insensitive metacontrol of these decision-making strategies.

NFC reflects an individual’s intrinsic motivation for cognitively demanding activities^16^. Previous research has shown that individuals high in NFC need less monetary incentives to exert cognitive effort than individuals low in NFC: Higher NFC is associated with an increased willingness to spent time with a cognitively demanding activity in the absence of reward^17^ and high-NFC individuals need fewer additional rewards to prefer a high-effort task over a low-effort task^18^. In line with these findings, a recent study reported that NFC modulates how rewards affect effort expenditure for executive functions^19^. Specifically, while individuals low in NFC invested more cognitive effort in a high-reward condition compared to a low-reward condition of a task-switching paradigm, this reward-induced effect vanished for individuals high in NFC. This suggests that the adaptation of cognitive effort toward different payoffs is more pronounced when the intrinsic motivation to engage in cognitively effortful behavior is low.

Based on these findings of how the intrinsic motivation for cognitive effort modulates the reward-related upregulation of cognitive effort^19^, we expect that NFC explains individual differences in metacontrol of decision-making strategies. Specifically, individuals high in NFC should show less metacontrol of decision making (i.e. a weaker upregulation of model-based decision making when rewards are amplified) than individuals low in NFC. We therefore hypothesize that NFC negatively correlates with reward-related metacontrol of decision making. We tested this prediction in datasets from two studies (Ns = 126 and 205) employing a decision-making task that has been developed to dissociate model-free from model-based strategies^7,20^. This task has been adapted from previous sequential decision-making tasks^1,21^ but in contrast to its predecessors, in the task employed in our studies, more reliance on a model-based strategy pays off in terms of higher rewards.

## Results

### Study 1

**Figure 1:**
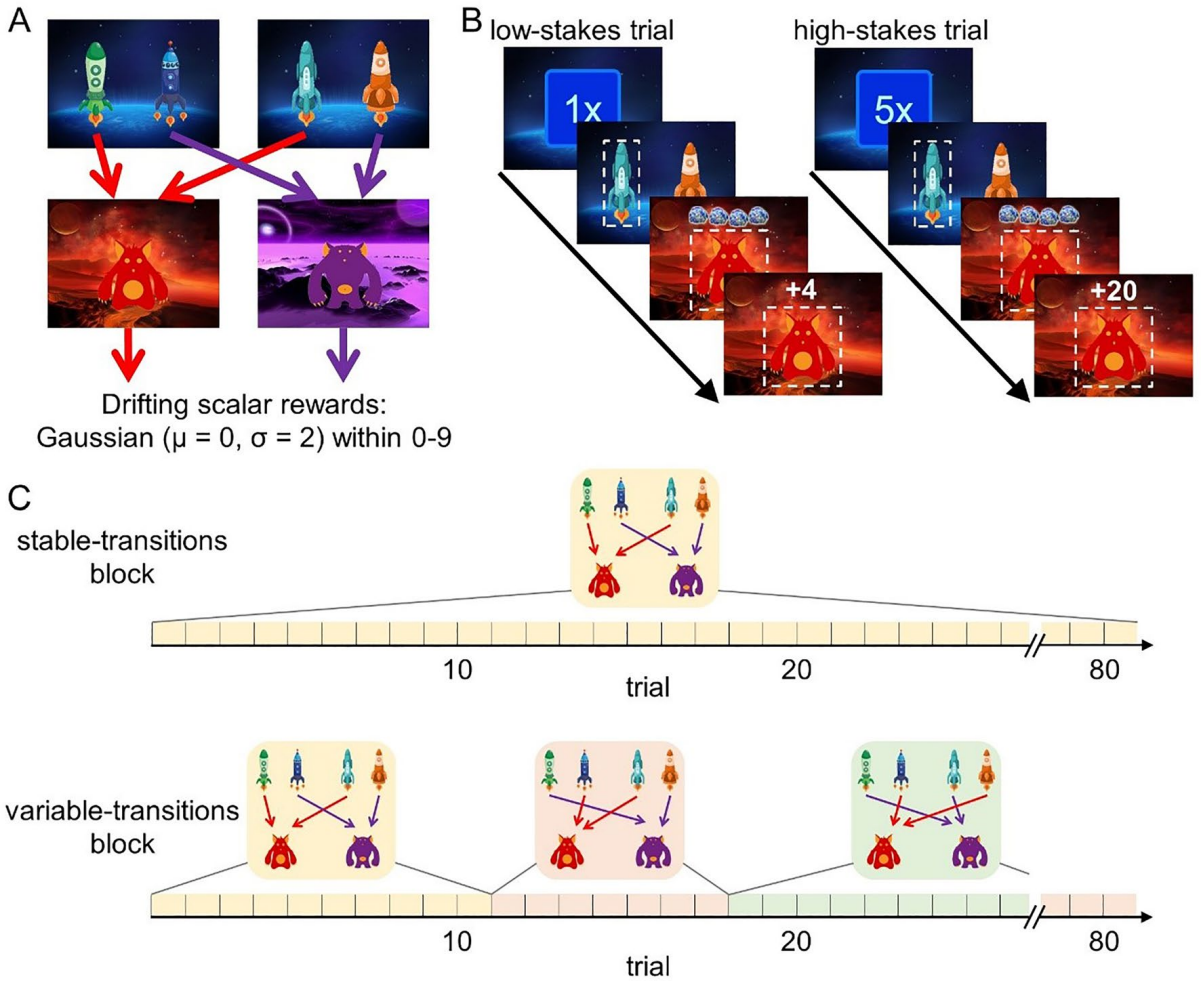
The sequential decision-making task. (**A**) Task transition structure. Each trial offered the choice between one pair of spaceships, both leading deterministically to one of two planets. At the planet, a reward was obtained and the amount of reward slowly drifted over the course of the task. (**B**) Trial structure. At the beginning of each trial, a stakes condition was cued. Low-stakes trials and high-stakes trials differed in how rewards were converted into points. (**C**) Transition conditions (only in Study 1). In stable-transitions blocks, the task transition structure remained unchanged throughout the block of 80 trials. In variable-transitions blocks, every 6–14 trials, the pair of spaceships in one first-stage state swapped their destination planets. This figure was reprinted from Ref.^9^ (license: https://creativecommons.org/licenses/by/4.0/).

Reliance on decision-making strategies was assessed with a sequential decision-making task that dissociates model-free and model-based decision making (Fig. 1; Refs.^9,20^) in a sample of 126 participants (76 female, age range 18–36 years). In this task, participants collect rewards (“space treasure”) by choosing between spaceships that travel to two different planets. The number of rewards available at these planets ranged between 0 and 9 and slowly drifted over the course of the task at both planets independently. Across trials, we manipulated how rewards were converted into points^7^. In *low-stakes trials*, participants received one point for every piece of space treasure; in *high-stakes trials,* participants received five points for every piece of space treasure (Fig. 1B). Moreover, we manipulated task complexity by imposing additional demands on structure learning in some trial blocks. In *stable-transitions blocks*, spaceships maintained their destinations throughout the entire block, while in *variable-transitions blocks*, one pair of spaceships switched their destinations every 6–14 trials (Fig. 1C). By means of this manipulation, we were able to assess metacontrol at two different levels of effort required for using a model-based strategy.

We used an established hybrid reinforcement-learning model^1,9,22^ to determine individual reliance on model-based decision making in the decision-making task. This model integrates model-free reward expectations *Q*_*MF*_(*s,a*) attained by simple temporal-difference learning and model-based reward expectations *Q*_*MB*_(*s,a*) based on weighting reward expectations for the final states according to the probability of getting there. The model-based weight ω (ranging between 0 and 1) reflects the relative influence of the model-based learner with higher values of ω representing relatively more reliance on model-based decision making. Our model includes four model-based weights, one for low-stakes trials and one for high-stakes trials in both stable-transitions blocks and variable-transitions blocks. Following the definition of metacontrol as the regulation of decision-making strategies toward changing situational demand and in line with other studies investigating individual differences in metacontrol^9–11^, we defined metacontrol as the difference between the model-based weight for high-stakes trials and the model-based weight for low-stakes trials, such that a higher difference value reflects a stronger upregulation of model-based decision making.

#### Metacontrol of decision making

**Figure 2:**
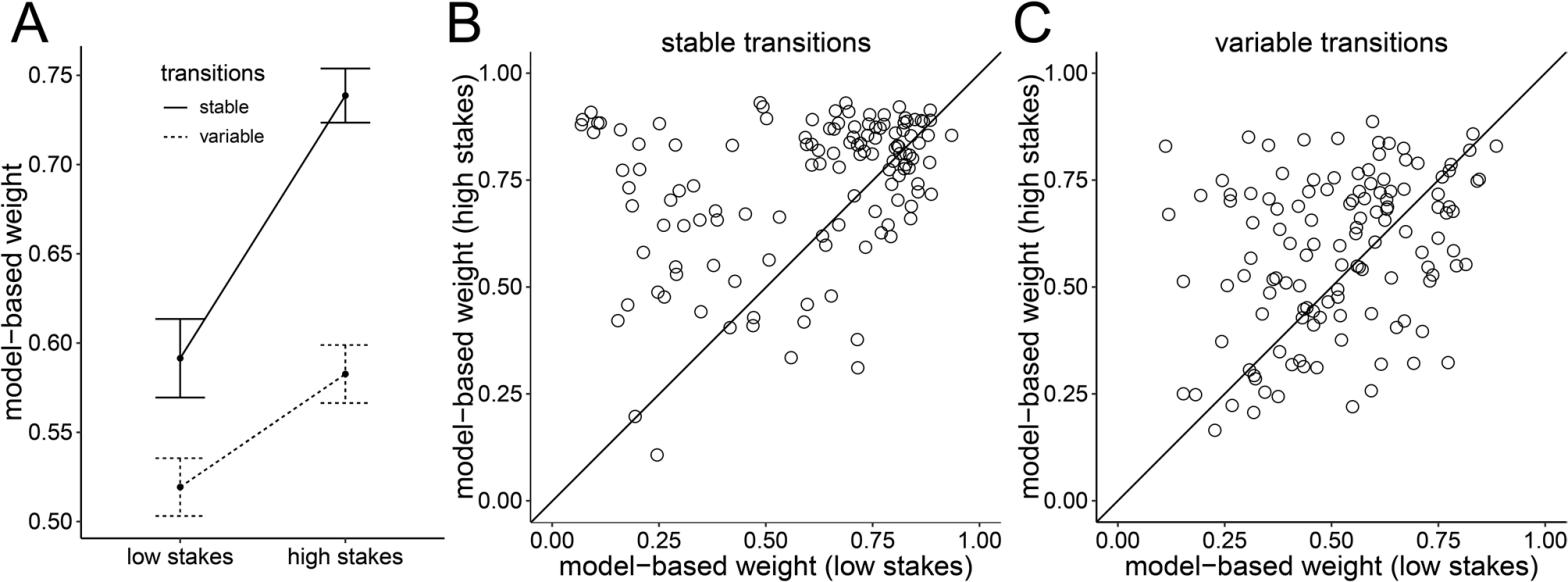
Metacontrol of decision making in Study 1. (**A**) Mean model-based weights. Error bars represent standard error of the mean. (**B**,**C**) Individual differences in model-based weights. Plots show model-based weights in high-stakes trials (y-axis) against model-based weights in low-stakes trials (x-axis) for stable-transitions blocks (**B**) and variable-transitions blocks (**C**). Points on the identity line represent individuals that showed no adaptation of model-based weights toward stakes conditions. Points above (below) the identity line represent individuals that showed higher (lower) model-based weights in high-stakes trials compared to low-stakes trials.

We found increased model-based weights for high-stakes trials compared to low-stakes trials (Fig. 2A), both in stable-transitions blocks (BF_10_ = 6.9 × 10^6^) and in variable-transitions blocks (BF_10_ = 19.3). In line with results previously reported in a subsample of our participants and in other studies^7,9^, this indicates that participants increased reliance on model-based decision making when rewards were amplified. Moreover, there were reduced model-based weights in variable-transitions blocks compared to stable-transitions blocks, both for low-stakes trials (BF_10_ = 29.4) and for high-stakes trials (BF_10_ = 2.0 × 10^13^). Thus, participants showed less model-based decision making during blocks with additional structure learning demands.

To better understand how the reliance on model-based decision making affects task behavior, we analyzed differences in task performance between the experimental conditions. Task performance was assessed as number of collected rewards corrected for the baseline of the average number of rewards available. In stable-transitions blocks, task performance was increased in high-stakes trials compared to low-stakes trials (*M*s = 0.62 vs. 0.32, BF_10_ = 198,883,045). Similarly, task performance was higher in high-stakes trials than in low-stakes trials in variable-transitions blocks (*M*s = 0.41 vs. 0.25, BF_10_ = 5065). This shows that the differences in model-based decision making reported above are mirrored by similar increases in task performance.

#### Need for cognition and metacontrol

**Figure 3:**
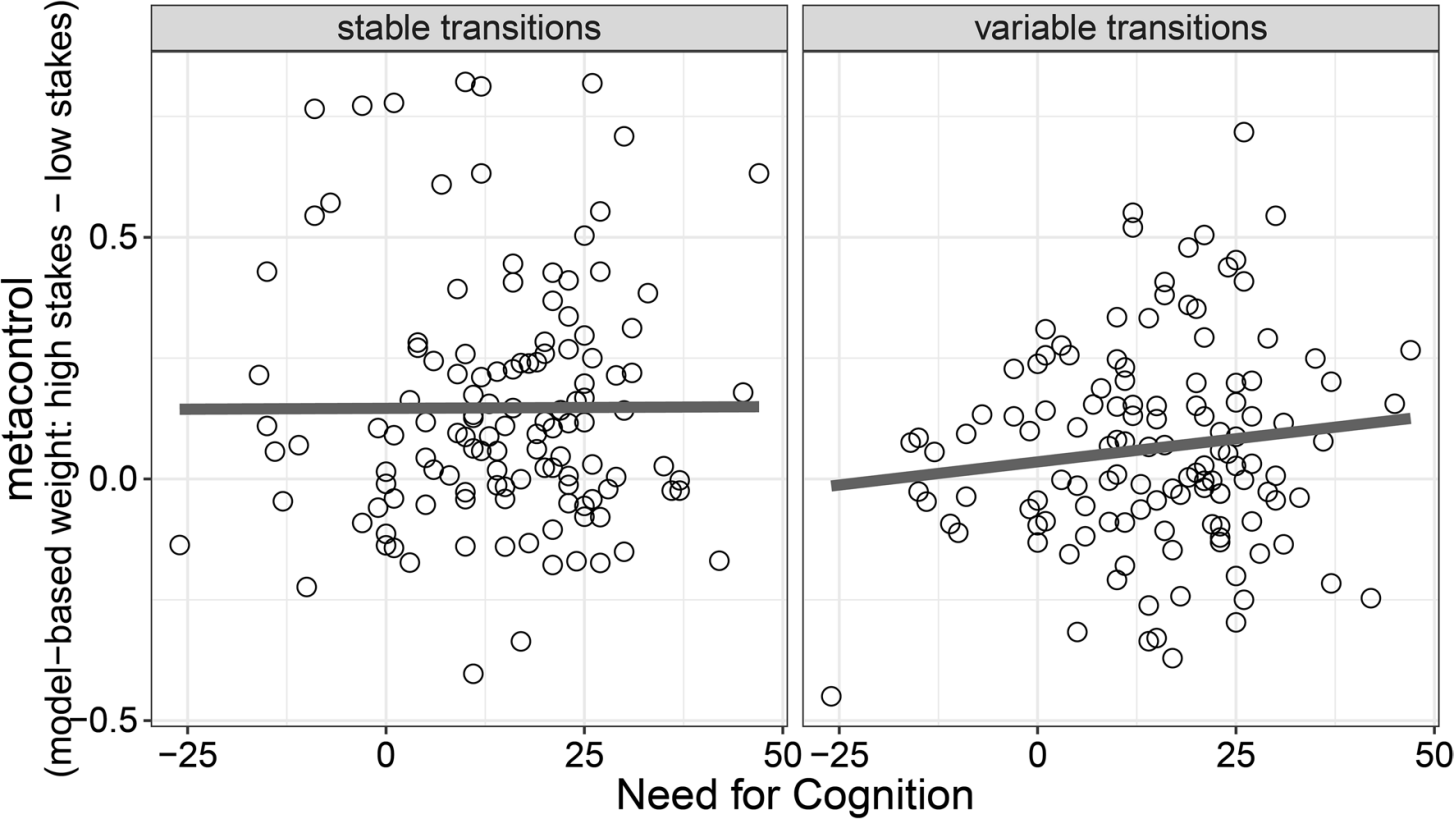
Relationship between NFC (x-axis) and metacontrol of decision making (y-axis) in Study 1.

We found considerable individual differences in how strongly participants adapted decision-making strategies in response to amplified rewards with some participants showing almost no difference in model-based decision making as a function of reward magnitude (points close to the identity line in Fig. 2B,C) whereas other participants showed pronounced differences in model-based decision making between low-stakes and high-stakes trials (points far away from the identity line in Fig. 2B,C). Therefore, we next analyzed whether NFC (as assessed with the German NFC short-scale^23^) explains individual differences in metacontrol of decision-making. We found moderate evidence against a correlation between NFC and metacontrol (Fig. 3) for both stable-transitions blocks (*r* = 0.00, BF_10_ = 0.11) and variable-transitions blocks (*r* = 0.12, BF_10_ = 0.28). Based on findings showing stronger reward-induced increases in executive function performance for individuals low in NFC^19^, we also tested the hypothesis of a negative correlation between NFC and metacontrol. We found moderate evidence against a negative correlation in stable-transitions block (BF_10_ = 0.11) and strong evidence against a negative correlation in variable-transitions blocks (BF_10_ = 0.05). These results were largely unaffected by different prior assumptions about the population correlation (see Supplementary Fig. S2 in the supplementary information). These findings speak against the idea that individuals low in NFC show more metacontrol of decision making.

Similarly, we found moderate evidence against a correlation between NFC and the stakes-related increase in task performance in stable-transitions blocks (*r* = − 0.10, two-sided BF_10_ = 0.21) and in variable-transitions blocks (*r* = 0.05, two-sided BF_10_ = 0.13). Thus, individuals low in NFC did not more strongly increase task performance as a function of reward magnitude and this suggests that they also do not differ in the adaptation of other decision-making processes that contribute to performance in this task.

#### Need for cognition and model-based decision making

We also investigated whether NFC explains individual differences in model-based decision making. There was moderate evidence against a correlation between NFC and model-based weights in low-stakes trials for both stable-transitions blocks (*r* = 0.09, BF_10_ = 0.18) and variable transitions-blocks (*r* = 0.07, BF_10_ = 0.15). There was inconclusive evidence regarding a correlation between NFC and model-based weights for high-stakes trials in both stable-transitions blocks (*r* = 0.13, BF_10_ = 0.34) and variable-transitions blocks (*r* = 0.21, BF_10_ = 1.73). Thus, our results do not show that NFC explains individual differences in model-based decision making.

Similarly, there was moderate evidence against a correlation between NFC and task performance for both low-stakes (*r* = 0.11, BF_10_ = 0.24) and high-stakes trials (*r* = 0.01, BF_10_ = 0.11) in stable-transitions blocks as well as for low-stakes trials in variable-transitions blocks (*r* = 0.05, BF_10_ = 0.13). There was inconclusive evidence regarding a correlation between task performance and NFC in high-stakes trials in variable-transitions blocks (*r* = 0.14, BF_10_ = 0.38). Thus, NFC does not seem to be related to performance in the decision-making task.

### Study 2

To scrutinize the unexpected findings in Study 1, we tried to replicate these findings in an independent, larger sample (N = 205, 149 female, age range 18–32 years). This sample was assessed as part of a more comprehensive study investigating the relationship between cognitive effort expenditure and ADHD personality traits in a student sample. For our purposes, we will focus our analysis here only on the variables corresponding to Study 1. We used an adapted version of the decision-making task that was employed in Study 1. Similar to Study 1, we manipulated how rewards were converted into points, with each piece of reward being worth one point in low-stakes trials and being worth five points in high-stakes trials. In contrast to Study 1, the spaceships kept their destination planets throughout the entire task, i.e., the complete task corresponded to the stable-transitions condition from Study 1.

#### Metacontrol of decision making

**Figure 4:**
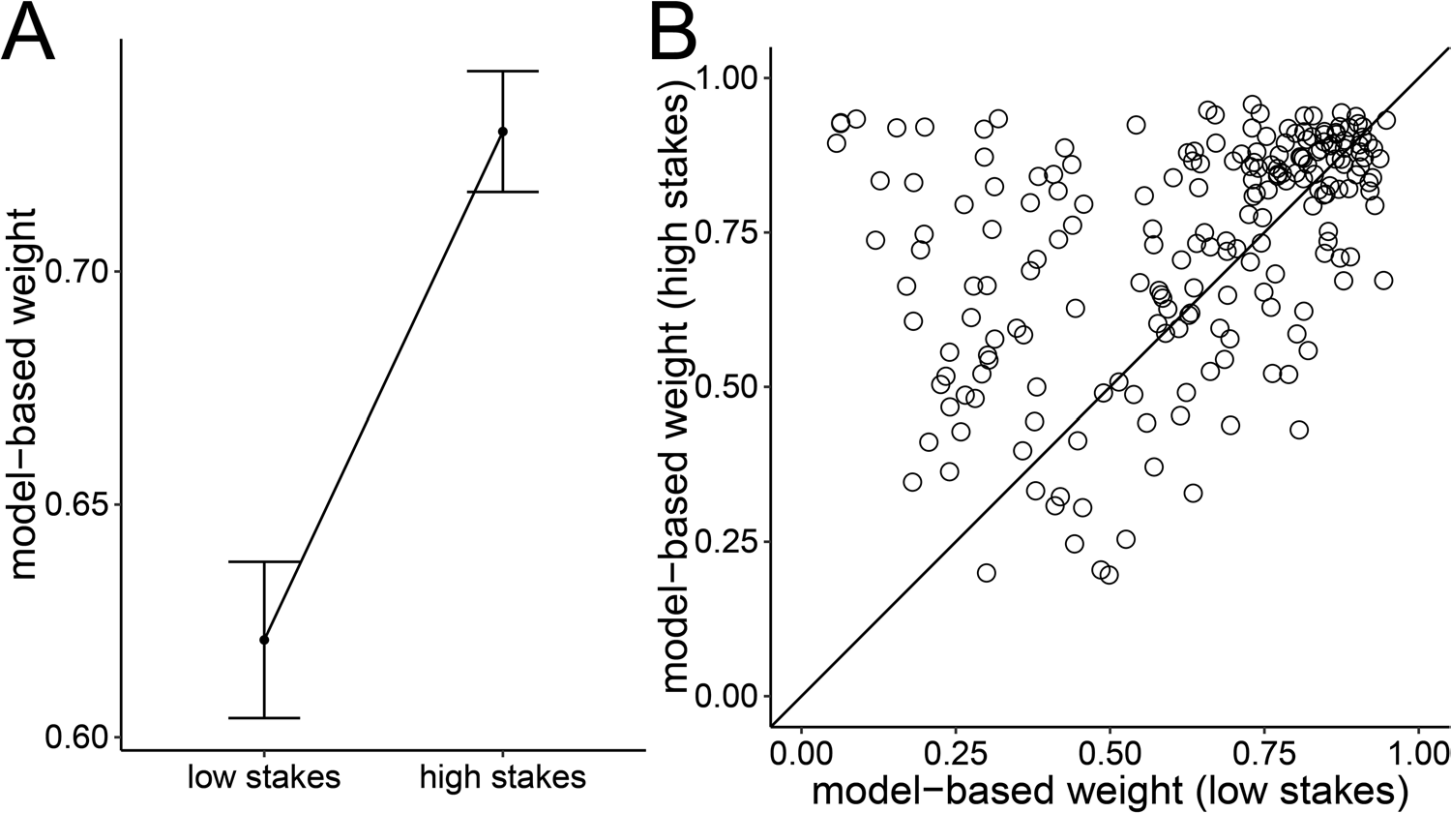
Metacontrol of decision making in Study 2. (**A**) Mean model-based weights. Error bars represent standard error of the mean. (**B**) Individual differences in model-based weights. Plots show model-based weights in high-stakes trials (y-axis) against model-based weights in low-stakes trials (x-axis). Points on the identity line represent individuals that showed no adaptation of model-based weights toward stakes conditions. Points above (below) the identity line represent individuals that showed higher (lower) model-based weights in high-stakes trials compared to low-stakes trials.

We found increased model-based weights in high-stakes trials compared to low-stakes trials (BF_10_ = 2.3 × 10^7^, Fig. 4A). Moreover, task performance was increased in high-stakes trials compared to low-stakes trials (*M*s = 0.31 vs. 0.18, BF_10_ = 24,567). Thus, similar to Study 1, participants showed metacontrol of decision making and relied more on model-based decision making when rewards were amplified. This increased reliance on model-based decision making was associated with better performance in the task.

#### Need for cognition and metacontrol

**Figure 5:**
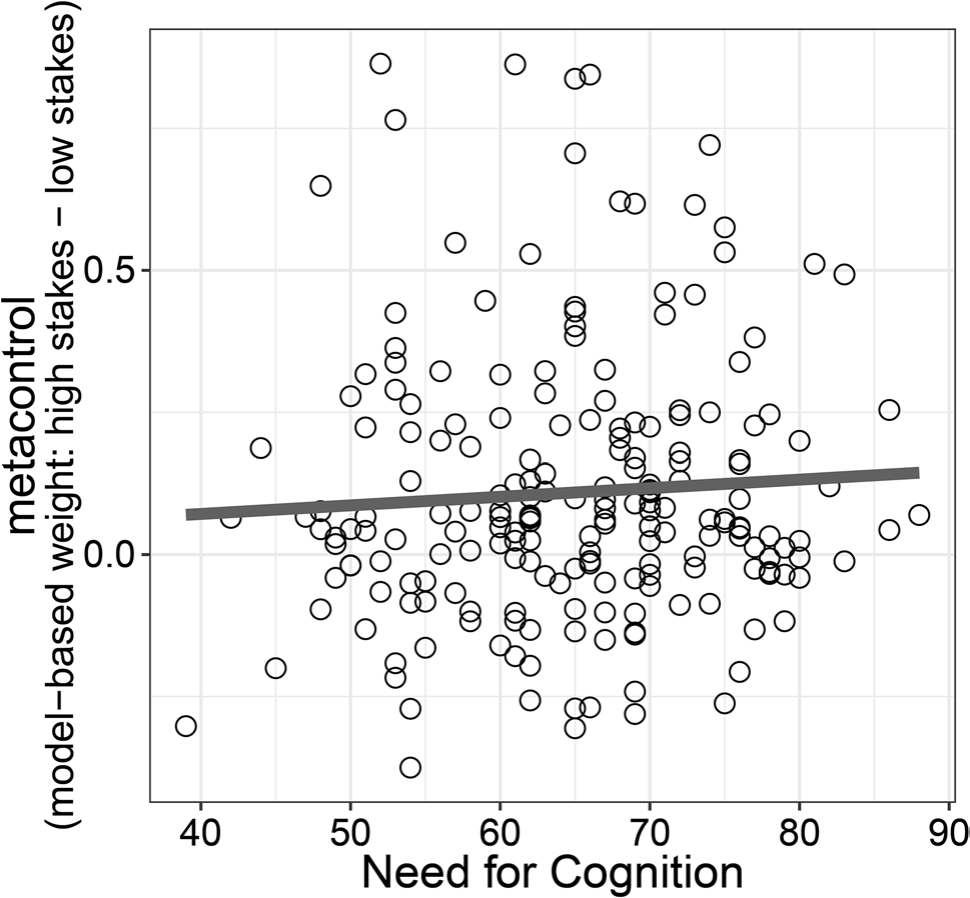
Relationship between NFC (x-axis) and metacontrol of decision making (y-axis) in Study 1.

As in Study 1, we observed considerable individual differences in metacontrol of decision making with some participants showing no adaptation of model-based weights toward the different stakes conditions while other participants showed a strong upregulation of model-based decision making when rewards were amplified (Fig. 4B). We found moderate evidence against a correlation between NFC (as assessed with the English NFC short-scale^16,24^) and metacontrol (*r* = 0.06, BF_10_ = 0.24, Fig. 5). Moreover, we found strong evidence against a negative correlation between NFC and metacontrol (BF_10_ = 0.09). Consistent with our findings in Study 1, these results speak against a role of NFC in explaining individual differences in metacontrol of decision making. Bayes Factor robustness checks indicated that these findings were largely unaffected by different prior assumptions (see Supplementary Fig. S3 in the supplementary information).

We found moderate evidence against a correlation between NFC and the stakes-related increase in task performance (*r* = − 0.11, BF_10_ = 0.27). Thus, individuals low in NFC did not seem to increase task performance more strongly when rewards were amplified.

#### Need for cognition and model-based decision making

We found inconclusive evidence regarding a correlation between NFC and model-based weights in low-stakes trials (*r* = 0.10, BF_10_ = 0.47). In contrast to our findings in Study 1, we found strong evidence for a correlation between NFC and model-based weights in high-stakes trials (*r* = 0.21, BF_10_ = 15).

There was a positive correlation between NFC and task performance in low-stakes trials (*r* = 0.22, BF_10_ = 10) and inconclusive evidence regarding a correlation between NFC and task performance in high-stakes trials (*r* = 0.14, BF_10_ = 0.70). Thus, different from Study 1, the results from Study 2 partly indicate that higher NFC is associated with more model-based decision making or better task performance.

## Discussion

We investigated individual differences in metacontrol of decision making in two independent studies. Based on previous research showing that individuals high in NFC show less regulation of cognitive effort in response to different incentive sizes, we expected metacontrol to be reduced with higher NFC. In contrast to our expectations, NFC did not explain individual differences in metacontrol. That is, individuals low and high in NFC similarly increased their reliance on the more accurate but more effortful model-based decision-making strategy when rewards were amplified. These findings show that the effect of incentives on the engagement of effortful decision-making strategies is largely independent of the intrinsic motivation for engaging in cognitively effortful activities. By means of a Bayes Factor analysis, we were indeed able to show that the data support the independence of NFC and metacontrol and that the lack of an association is not merely due to low statistical power (in which case the Bayes Factors would support neither independence nor a correlation between the variables; cf.^25^). Mirroring the independence of NFC and metacontrol of decision making, participants low in NFC also did not show more adaptation of general task performance as a function of reward magnitude.

Our findings suggest that NFC may have a differential role for the expenditure of cognitive effort in decision-making and executive functions. In a study by Sandra and Otto^19^, participants low in NFC showed a stronger ramp-up of cognitive effort in a task-switching paradigm when rewards were amplified than participants high in NFC. Although model-based decision making relies at least partly on executive functions^4,5^, we did not observe a similar relationship between NFC and cognitive effort expenditure during decision making. This could point to a potential process specificity regarding the role of NFC in the regulation of cognitive effort.

Furthermore, we obtained inconsistent evidence of the relationship between NFC and model-based decision making. While the results from Study 2 suggest that high-NFC individuals show more model-based decision making than low-NFC individuals under high-stakes conditions, this was less evident in Study 1. This does in parts parallel findings in the domain of multi-attribute decision making where NFC was not associated with individual tendencies to engage in more complex weighting of different attributes instead of relying on a more simple take-the-best heuristic^26^. Taken together, NFC does not seem to be a reliable predictor for strategy use in decision making that generalizes across tasks.

While NFC generally has been associated with more cognitively effortful modes of information processing^16^, a recent study points to no relationship between NFC and basic executive functions^27^ which largely mirrors the results from our studies. A potential explanation for these inconsistent findings could be that our decision-making task—like most paradigms in experimental psychology—poses strong situational affordances and thus might reduce interindividual variability, making it more challenging to use in correlational research^28^.

A possible limitation of our studies is that they were based on student samples with a distribution of NFC scores shifted above the scale mean. This restricted range could have attenuated the association between NFC and metacontrol and future studies should rely on more representative samples. However, this sample characteristic equally holds for the study by Sandra and Otto^19^ and we therefore think it is unlikely that this factor explains the absence of a correlation in our two studies.

Other studies reporting associations between model-based decision making or its metacontrol and personality traits^11,13^ based their findings on considerably larger sample sizes than in our two studies presented here. However, in contrast to these previous studies we did not test participants via online crowdsourcing platforms but in lab-based studies, potentially making up for the lower sample size with a more controlled, less noise-prone experimental setup. Moreover, we additionally fitted the data with a hierarchical Bayesian version of the reinforcement-learning model (see Supplementary Information) which can account for uncertainty of parameter estimates. In this analysis, we also found moderate to strong evidence against a negative correlation between NFC and metacontrol, consistent with the results reported in the main analysis.

The stakes manipulation used in our task (factor 1 versus factor 5) was comparable in size to the stakes manipulation in the study by Sandra and Otto^19^ (1 cent versus 5 cent). In contrast to our experimental design, where the stakes condition was assigned to each trial randomly, Sandra and Otto^19^ employed a blockwise stakes manipulation, keeping the stakes condition constant for several trials in a row. Thus, in our two studies, individual differences in the willingness to exert cognitive effort might have been attenuated by individual differences in the ability to adapt cognitive effort dynamically and immediately. However, since young adults can adapt behavioral performance in cognitive control tasks rapidly to changes in the reward structure^29^, we think that this difference in task design is unlikely to explain the different patterns in our study and the study by Sandra and Otto^19^. Further research is needed to better understand the temporal dynamics of metacontrol.

In conclusion, we found that NFC does not account for individual differences in metacontrol of decision making. That is, individuals low and high in NFC equally adapted their reliance on a more accurate but more effortful strategy when rewards were amplified. While some of our findings suggest that high-NFC individuals exert more cognitive effort during decision making, this relationship was not observed consistently in both studies and encourages more research on the role of personality traits in reinforcement learning.

## Methods

### Study 1

For Study 1, we report how we determined our sample size, all data exclusions, all manipulations, and all measures in the study^30^. The dataset and all analysis scripts can be found at osf.io/9wc4u.

#### Participants

128 participants took part in this study. Data from a subset of this sample (N = 63) have been collected as part of a different study^9^ and for these participants additional measures such as cognitive control and processing speed were assessed that are not reported here. For the purpose of the current study we recruited additional participants based on considerations that it would take around 120 participants to detect a true correlation of r = 0.25 with a power of 1 − β = 0.80. We excluded two participants from data analysis due to the following reasons: missing responses in more than 20% of trials in the decision-making task (1) and missing values in the NFC scale (1). Thus, the effective sample consisted of 126 participants (76 female, age range 18–36 years, mean age = 23.4 years). All participants gave informed written consent and received either monetary compensation (5€/h) or course credit for their participation, as well as an additional monetary compensation related to their performance in the decision-making task (10 cents for every 60 points in the decision-making task). The ethics committee of Technische Universität Dresden approved the study and all research was performed in accordance with the relevant guidelines and regulations.

#### Decision-making task

We employed a sequential decision-making task that had been developed to dissociate model-free and model-based decision making^7,9,20^. In contrast to previous tasks it has been adapted from Refs.^1,21^, more reliance on model-based decision making pays off in terms of higher rewards in the task employed here.

Each trial started with an intertrial interval (black screen, 750 ms) and the presentation of a stakes cue (1000 ms) that signal how the rewards earned in this trial were converted into points. Both stakes cues (low-stakes cue = “1×” and high-stakes cue = “5×”) were assigned with equal probability to trials. After this, one of two first-stage states was presented (3000 ms) with two spaceships displayed side by side (an orange and a turquoise spaceship in one first-stage state, and a green and a blue spaceship in the other first-stage state; all spaceships were displayed equally often on the left or the right side). Participants selected the left or the right spaceship using the keys F and J on a standard computer keyboard and after a choice was made, the respective spaceship was highlighted for the remaining time of the presentation of the first-stage state. Subsequently, one of two second-stage states (a red or a purple planet with an alien) was presented and the second-stage state was deterministically determined by the choice of the spaceship. For each pair of spaceships, there was always one spaceship leading to the red planet and the other spaceship leading to the purple planet. In stable-transitions blocks, the mapping from spaceships to planets was held constant throughout the block, whereas in variable-transitions blocks, every 6–14 trials, one of the two pairs of spaceships switched their destination planets. Participants had 2000 ms to respond to the second-stage state by pressing the space bar. After this response window, the amount of rewards available at this planet and points received in this trial was shown. Rewards available at both planets were based on two independent Gaussian random walks (mean = 0, standard deviation = 2, reflecting boundaries at 0 and 9, values were rounded to integers). During the task, a total point count was displayed in the top-right corner of the screen.

If no response was given during the first-stage state or the second-stage state within the respective response window, the trial was canceled, no reward was given and the task proceeded with the next trial. Trials with missing responses at the first-stage state were not included in the analysis (1% of all trials). Different to previous studies with this task, we included trials with missing responses at the second-stage state because these trials could be potentially informative for updates of the transition structure.

The task consisted of 320 trials, grouped into four blocks of 80 trials. Between blocks, the transition condition (stable vs. variable) was alternating and participants were informed at the beginning of each block about the transition condition for the upcoming trials.

In order to decrease variability between participants due to random variations in the task, we kept the reward trajectories and the sequence of first-stage states identical for all participants. The assignment of stakes conditions to trials and of transition conditions to blocks was counterbalanced across participants.

Before starting with the sequential decision-making task, participants received a detailed instruction about the nature of the reward distribution, the transition structure and the stakes manipulation. To ensure their understanding of the task, participants had to select the spaceship leading to a planet (with 10 consecutive correct choices necessary for each planet to proceed) and to specify the number of points given a number of rewards and a stakes cue (with 10 consecutive correct answers necessary to proceed). Moreover, they performed 20 training trials for the stable-transitions condition and 20 training trials for the variable-transitions condition.

#### Reinforcement-learning model

The model-free learner holds reward expectations *Q*_*MF*_(*s*_*i*_,*a*_*i*_) for each action *a*_*i*_ and state *s*_*i*_ at the *i*-th stage of the task. At the beginning of the task, all reward expectations are set to 4.5 (reflecting the mean of the range of possible rewards). After each choice, all reward expectations are updated according to a temporal-difference learning rule:

$${\mathrm{Q}}_{\mathrm{MF}}\left({\mathrm{s}}_{i},{\mathrm{a}}_{\mathrm{i}}\right)\leftarrow {\mathrm{Q}}_{\mathrm{MF}}\left({\mathrm{s}}_{\mathrm{i}},{\mathrm{a}}_{\mathrm{i}}\right)+\alpha \times e\left({s}_{i},{a}_{i}\right)\times \delta .$$

Here, α is the reward learning rate (bounded between 0 and 1) reflecting how quickly new experiences are integrated into reward expectations. The eligibility trace *e*(*s*_*i*_*,a*_*i*_) is set to 0 for all combinations of *s*_*i*_ and *a*_*i*_ at the beginning of a trial; before updating reward expectations, the eligibility trace for the immediately preceding state-action pair (*s*_*i′*_, *a*_*i′*_) is set to 1 and after the update, all eligibility traces are decayed by the eligibility trace decay parameter λ (bounded between 0 and 1). The reward prediction error δ reflects the discrepancy between experienced and expected reward and is computed as

$$\updelta ={\mathrm{r}+\mathrm{Q}}_{\mathrm{MF}}\left({s}_{{i}^{^{\prime}}+1},{\mathrm{a}}_{{i}^{^{\prime}}+1}\right)-{\mathrm{Q}}_{\mathrm{MF}}\left({\mathrm{s}}_{{\mathrm{i}}^{\mathrm{^{\prime}}}},{\mathrm{a}}_{{\mathrm{i}}^{\mathrm{^{\prime}}}}\right),$$

where *r* is the immediate reward obtained after a choice (note that *r* is always 0 after first-stage choices) and *Q*_*MF*_(*s*_*i′*+1_,*a*_*i′*+1_) is the reward expectation associated with the subsequent action *a*_*i′*+1_ in the new state *s*_*i′*+1_ (note that *Q*_*MF*_(*s*_*i′*+1_,*a*_*i′*+1_) is always 0 after second-stage choices because the states that offer rewards are terminal).

The model-based learner maintains a model of the task structure represented by a transition matrix *T*(*s*_2_*|s*_1_, *a*_1_) that holds probabilities for moving to a second-stage state given an action and a first-stage state. At the beginning of the task, all transition probabilities are 0.5 and after observing a transition to a second-stage state, these probabilities are updated according to

$$T\left({s}_{2}|{s}_{1},{a}_{1}\right)\leftarrow T\left({s}_{2}|{s}_{1},{a}_{1}\right)+\eta \times {\delta }^{SPE},$$

$$T\left({\neg s}_{2}|{s}_{1},{a}_{1}\right)\leftarrow T\left({\neg s}_{2}|{s}_{1},{a}_{1}\right)\times \left(1-\eta \right).$$

Here, η is the transition learning rate (bounded between 0 and 1) that reflects how quickly observations of transitions are integrated into the representation of the task structure. To ensure that the sum of transition probabilities stays 1, the probability for transitioning to the alternative second-stage state ⌐*s*_*2*_ needs to be adjusted. The state prediction error δ^SPE^ is computed as

$${\delta }^{SPE}=1-T\left({s}_{2}|{s}_{1},{a}_{1}\right).$$

It is possible to infer the second-stage state to which the alternative, not-chosen first-stage action would have led because both actions available in a first-stage state always lead to different second-stage states. Thus, the model-based learner also updates transition probabilities for the alternative action in the same way as it does for the actual actions, using a counterfactual transition learning rate η_CF_ (bounded between 0 and 1).

While the model-based reward expectations at the second stage are identical to the model-free reward expectations (because both reflect an estimate of the immediate reward), the model-based reward expectations at the first stage are computed as

$${Q}_{MB}\left({s}_{1},{a}_{1}\right)= \sum_{{s}_{2}}T\left({s}_{2}|{s}_{1},{a}_{1}\right){Q}_{MB}({s}_{2},{a}_{2}).$$

At the first stage, both model-free and model-based reward expectations are combined to an integrated reward expectation *Q*(*s*_1_,*a*_1_) with the model-based weight ω (bounded between 0 and 1) reflecting the relative influence of the model-based learner.

$$Q\left({s}_{1},{a}_{1}\right)=\left(1-\omega \right){Q}_{MF}\left({s}_{1},{a}_{1}\right)+\omega {Q}_{MB}\left({s}_{1},{a}_{1}\right).$$

Choice probabilities at the first stage are modeled by a softmax function:

$$P\left({a}_{1}|{s}_{1}\right)= \frac{\mathrm{exp}(\beta \left[Q\left({s}_{1},{a}_{1}\right)+\pi \cdot rep\left({a}_{1}\right)+\rho \cdot resp\left({a}_{1}\right)\right])}{{\sum }_{a{^{\prime}}}\mathrm{exp}(\beta [Q\left({s}_{1},a{^{\prime}}\right)+\pi \cdot rep(a{^{\prime}})+\rho \cdot resp(a{^{\prime}})])}.$$

Here, β is the inverse softmax temperature (left-bounded at 0) that reflects how consistently choices are guided by reward expectations. The choice stickiness π and the response stickiness ρ (both unbounded) capture perseveration (positive values) or switching (negative values) of choices (which stimulus was selected) or responses (which key was pressed) across trials. The indicator variables *rep*(*a*_1_) and *resp*(*a*_1_) are set to 1 if the same stimulus or the corresponding response key were selected in the previous trials (and are set to 0 otherwise).

#### Model-fitting procedure

We obtained individual maximum a posteriori parameter estimates using the mfit toolbox in Matlab^31^ with the following priors: Beta(2, 2) priors for α, λ, η, η_CF_ and ω; Normal(0,1) priors for π and ρ; a Gamma(3, 0.2) prior for β. To avoid local optima, the optimization procedure was started 100 times for each participant and we used the parameters of the run with the highest posterior probability. Model-based weights were estimated separately for low-stakes and high-stakes trials in both stable-transitions and variable-transitions blocks. Transition learning rates were estimated only for variable-transitions blocks and set to 1 during stable-transitions blocks. We fitted different versions of the model where the parameters λ, η, η_CF_, π and ρ were varied to be free or fixed parameters and we selected the best-fitting model version based on the Akaike Information Criterion (free parameters: λ, π, ρ; fixed parameters: η = η_CF_ = 1). As reported in Bolenz et al.^9^, the model shows good identifiability of parameters. Moreover, posterior predictive checks indicated that the model could capture individual differences in task behavior well (see Supplementary Information).

To better account for the uncertainty in the parameter estimates, we also fitted the data with a hierarchical Bayesian version of the reinforcement learning model (see Supplementary Information).

#### Task performance

Task performance was assessed as the number of rewards obtained in a trial (before any multiplication related to the stakes condition) subtracted by the baseline computed as the average number of rewards available at the two planets in the same trial. Thus, our measure of task performance reflects how much more rewards a participant had earned compared to the expected reward for a random decision maker. For each participant, we averaged task performance across all trials of the same experimental condition.

#### NFC scale

We assessed NFC with the German NFC short-scale^23^. This scale consists of 16 items that are recorded on a 7-point Likert scale, ranging from − 3 (“totally disagree”) to + 3 (“totally agree”). While the scale in our study actually ranged between 1 and 7, we transformed all values to the original scale for reasons of consistency with previous studies. In our sample, NFC scores ranged between − 26 and 47 (mean = 14.63, sd = 13.48). Internal consistency was α = 0.88.

#### Data analysis

For quantifying the evidence regarding our hypotheses, we computed Bayes Factors using the BayesFactors package in R^32^. Bayes Factors reflect how much more likely it is to observe some data under the assumption of the alternative hypothesis than under the assumption of the null hypothesis. Bayes Factors ranging between 3 and 10 are commonly interpreted as providing moderate evidence and Bayes Factors above 10 are interpreted as providing strong evidence for the alternative hypothesis. Conversely, Bayes Factors ranging between 1/3 and 1/10 are interpreted as providing moderate evidence and Bayes Factors below 1/10 are interpreted as providing strong evidence for the null hypothesis^33^.

For Bayes Factors concerning hypotheses about differences in means, we used JZS priors with scaling parameter r = √2/2^34^ and for Bayes Factors concerning hypotheses about correlations, we used stretched beta priors with scaling parameter κ = 1^35^ which assign equal prior probabilities to correlations between − 1 and 1. We also conducted Bayes Factor robustness checks for our primary analyses, varying κ between 0.01 and 1 (see Supplementary Fig. S2 in the supplementary information).

We performed a Bayesian analogue to power analysis for a two-sided correlation test (i.e., comparing evidence for a non-zero correlation and for no correlation) and a one-sided correlation test (i.e., comparing evidence for a negative correlation and for no correlation). For different true population correlations ρ in a population of N = 1000, we computed the proportion of 10,000 random samples of n = 126 for which a Bayes Factor would show at least moderate evidence for the null hypothesis or the alternative hypothesis. With our sample of N = 126 and in a two-sided correlation test, we would find at least moderate evidence with 80% probability for a non-zero correlation if the true population correlation is |ρ|≥ 0.31 and for no correlation if the true population correlation is |ρ|≤ 0.06. In a one-sided correlation test, we would find at least moderate evidence with 80% probability for a negative correlation if the true population correlation is ρ ≤ − 0.28 and for no correlation if the true population correlation is ρ ≥ − 0.02.

### Study 2

For Study 2, we report how we determined our sample size, all data exclusions and all manipulations^30^. The dataset and all analysis scripts can be found at osf.io/9wc4u.

#### Participants

214 participants took part in this study. Sample size was determined based on feasibility considerations and with respect to research questions regarding the relationship between cognitive effort expenditure and ADHD personality traits that were the focus of a more comprehensive study. Participants completed the decision-making task and the NFC scale as part of a larger task battery, the results of which will be reported elsewhere. We excluded nine participants from data analysis due to the following reasons: missing responses in more than 20% of trials in the decision-making task (1), key repetitions in more than 95% of trials in the decision-making task (1), no or incomplete recording of the decision-making task due to technical difficulties (3), duplicate assessments with the NFC scale (4). Thus, the effective sample consisted of 205 participants (149 female, age range 18–32 years, mean age = 22.0 years). All participants gave informed written consent and received monetary compensation ($25 or course credit as baseline, 22 cents for every 100 points in the decision-making task). The Human Research Ethics Committee at Concordia University approved the study and all research was performed in accordance with the relevant guidelines and regulations.

#### Decision-making task

We used a variant of the sequential decision-making task from study 1. Trials followed the same structure, but with a different pacing. The intertrial interval was presented for 300 ms, the stakes cue was presented for 800 ms, and both first-stage state and second-stage state were presented for 1500 ms each. The entire task consisted of 280 trials, equally distributed between low-stakes and high-stakes trials. There were no changes in the transition structure, so all spaceships kept their initial destination planets.

We excluded all trials from analysis in which no response was given during the presentation of either the first-stage state or the second-stage state (3% of all trials).

In order to decrease variability between participants due to random variations in the task, we created two independent trial sequences that determined reward trajectories and first-stage states and that were counterbalanced across participants. Within each trial sequences, the assignment of stakes conditions to trials was counterbalanced.

Before starting the task, participants received a similar task instruction as in study 1, apart from performing 25 training trials, all with a stable task structure.

#### Reinforcement-learning model

We adapted the reinforcement-learning model from study 1. Due to no changes in the transition structure in this version of the task, we set the transition matrix *T* to reflecting the true transition probabilities at the beginning of the task and did not model any updates of the transition matrix, thus abandoning the transition learning rate and the counterfactual transition learning rate as model parameters. Also, this model only contained two model-based weights (one for low-stakes trials and one for high-stakes trials).

#### Model-fitting procedure

We used the same model-fitting procedure as in study 1 with the following exceptions: Only two model-based weights were fitted for each participant, one for low-stakes trials and one for high-stakes trials. We fitted different versions of the model where the parameters λ, π and ρ were varied to be free or fixed parameters and we selected the best-fitting model version based on the Akaike Information Criterion (free parameters: π, ρ; fixed parameter: λ = 0). Posterior predictive checks indicated that the model could capture individual differences in task behavior well (see supplementary information). Again, we also fitted the data with a hierarchical Bayesian version of the reinforcement learning model (see Supplementary Information).

#### NFC scale

We assessed NFC with the English NFC short-scale^16,24^. This scale consists of 18 items that are recorded on a 5-point Likert scale, ranging from 1 (“extremely uncharacteristic”) to 5 (“extremely characteristic”). In our sample, NFC scores ranged between 39 and 88 (mean = 64.81, sd = 9.68). Internal consistency was α = 0.84.

#### Data analysis

Different from Study 1, we used a scaling parameter κ = 1/3 for the stretched beta priors for Bayes Factors concerning hypotheses about correlations. Thus, more prior weight was given to correlation coefficients closer to 0, reflecting our findings in Study 1. We also conducted Bayes Factor robustness checks for our primary analyses, varying κ between 0.01 and 1 (see Supplementary Fig. S3 in the supplementary information).

We performed a Bayesian analogue to power analysis similar to Study 1. With our sample of N = 205 and in a two-sided correlation test, we would find at least moderate evidence with 80% probability for a non-zero correlation if the true population correlation is |ρ|≥ 0.23 and for no correlation if the true population correlation is |ρ|≤ 0.01. In a one-sided correlation test, we would find at least moderate evidence with 80% probability for a negative correlation if the true population correlation is ρ ≤ − 0.21 and for no correlation if the true population correlation is ρ ≥ − 0.001.

## Supplementary Information

Supplementary Information.
