# Supplementary material for: Need for cognition does not account for individual differences in metacontrol of decision making
Source: Sci Rep. 2022 May 17;12:8240. doi: 10.1038/s41598-022-12341-y (PMC9114337; doi:10.1038/s41598-022-12341-y)
Supplement: Supplementary file 1 — Supplementary Information. [file 41598_2022_12341_MOESM1_ESM.docx]

**Need for cognition does not account for individual differences in metacontrol of decision making**

**Supplementary information**

# Posterior predictive checks

To assess how good the reinforcement-learning model can capture individual differences in task behavior, we simulated choice data with the generative version of the reinforcement-learning model. For each participant, simulations were carried out 50 times with the best-fitting individual parameter estimates and the same task sequence as encountered by the participant. For each participant, we averaged task performance across all simulation runs. In both studies, we found a high correspondence between the simulated task performance and the empirically observed task performance (*r* = 0.86 for Study 1 and *r* = 0.81 for Study 2; cf. Figure S1), indicating that our model can capture the individual differences in task behavior well.

# Hierarchical Bayesian model fitting

To better account for the uncertainty of the parameter estimates, we additionally fitted the data with a hierarchical Bayesian version of the reinforcement-learning model. This approach has also been recommended to increase parameter reliability due to the partial pooling of parameter estimates [1]. Instead of outputting point estimates, the hierarchical model approximates probability density functions for every parameter and higher uncertainty of a parameter results in broader density functions.

Every participant-level parameter was modeled as being drawn from a group-level distribution with Normal(0, 1) priors for all group-level means and Normal(0, 2) priors for all group-level standard deviations. Bounded parameters (α, ω) were modeled in logit space but re-transformed to normal space for all subsequent analysis. We used the same model versions for the data of Study 1 and Study 2 as reported in the main analysis with the exception that for Study 1, λ was fixed to 1 due to better model convergence.

The models were fit using Stan via the RStan package [2]. We ran four independent chains of the Markov Chain Monte Carlo (MCMC) procedure with 8000 iterations each and discarded the first 4000 iterations of each chain as warm-up. We assessed the convergence of all chains via the Gelman-Rubin statistic ($\hat{R}$ < 1.1) for all parameters and ensured that bulk and tail effective sample size was sufficiently high (> 400) for all analyzed parameters.

To quantify metacontrol, the difference between high-stakes and low-stakes model-based weights (participant level) was calculated for every MCMC iteration. For computing the correlation with an external variable (NFC), the correlation between NFC and the model parameters in every MCMC iteration were computed, resulting in a probability density function for the correlation. Bayes Factors were computed using the Savage-Dickey method [3] with the same prior assumptions as in the main analysis.

For the data of Study 1, we found strong evidence against a correlation between NFC and metacontrol in the stable-transitions blocks (BF_10_ = 0.0996) and moderate evidence against a correlation in the variable-transitions blocks (BF_10_ = 0.13). When conducting one-sided tests, we found strong evidence against a negative correlation in both stable-transitions blocks (BF_10_ = 0.09) and variable-transitions blocks (BF_10_ = 0.06).

For the data of Study 2, we found moderate evidence against a correlation between NFC and metacontrol (BF_10_ = 0.13). When conducting a one-sided test, we found moderate evidence against a negative correlation (BF_10_ = 0.101).

In conclusion, the hierarchical model fitting corroborates our main analyses by showing that individuals low in NFC do not show more metacontrol of model-based decision making.

# Supplementary references

1. Brown, V. M., Chen, J., Gillan, C. M. & Price, R. B. Improving the reliability of computational analyses: Model-based planning and its relationship with compulsivity. *Biological Psychiatry: Cognitive Neuroscience and Neuroimaging* (2020) doi:10.1016/j.bpsc.2019.12.019.

2. Stan Development Team. *RStan: the R interface to Stan*. (2018).

3. Wagenmakers, E.-J., Lodewyckx, T., Kuriyal, H. & Grasman, R. Bayesian hypothesis testing for psychologists: A tutorial on the Savage–Dickey method. *Cognitive Psychology* **60**, 158–189 (2010).

| **Figure S1**  *Posterior predictive checks for Study 1 (A) and Study 2 (B). For each participant, the empirically observed task performance is plotted against the task performance of a simulated agent with the participant’s best-fitting parameter values. The diagonal represents the identity line and the closer a point is to the identity line, the better the model was able to capture the participant’s behavior.* |
| --- |
| 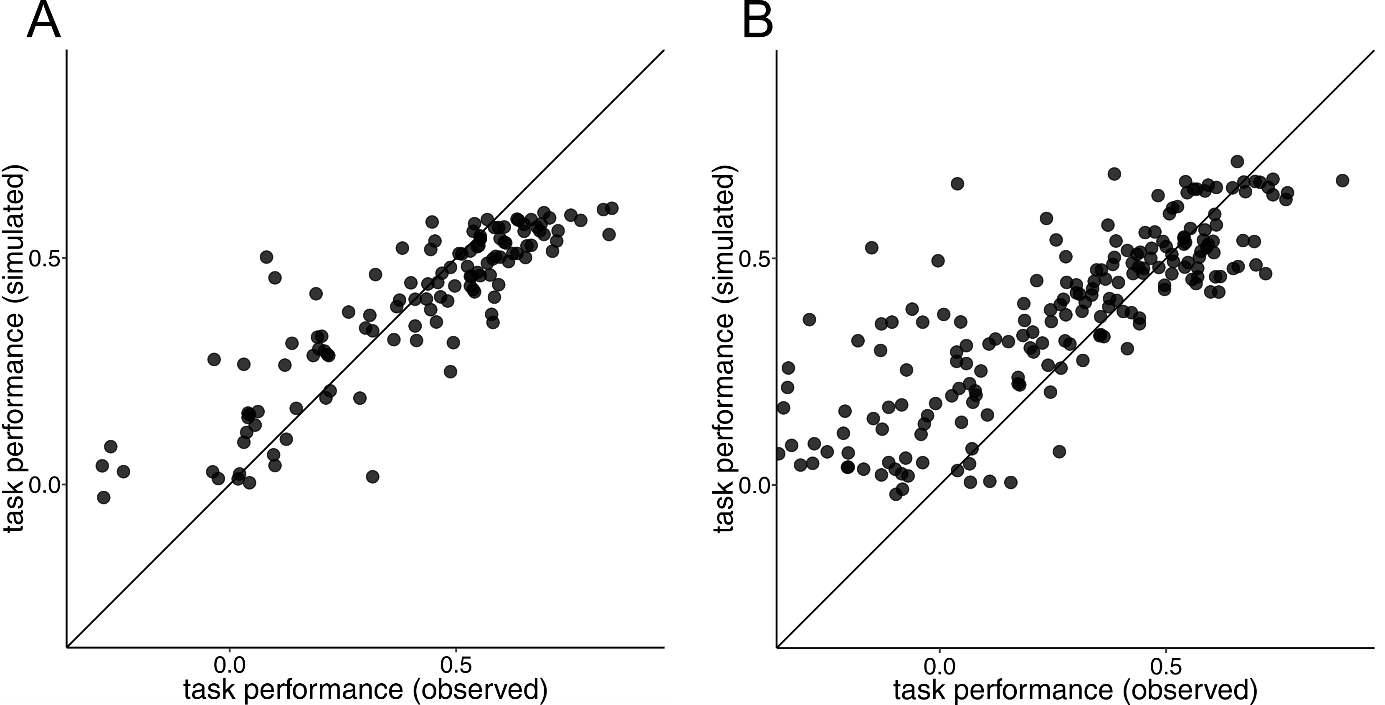 |

| **Figure S2**  *Bayes Factor robustness checks for the analyses of correlations between NFC and metacontrol in Study 1. Two-sided tests compare evidence for a non-zero correlation and for no correlation; one-sided tests compare evidence for a negative correlation and for no correlation. Plots show Bayes Factors for different values of the scaling parameter κ (ranging between 0.01 and 1). For κ = 1, all population correlations between -1 and 1 are equally likely a priori and with smaller values of κ, correlations near zero become more likely a priori than correlations close to -1 or 1. Highlighted are the scaling value used in this study (κ = 1, red) and the scaling value implemented as a default in the BayesFactor package (κ = 1/3, gray)* |  |
| --- | --- |
| **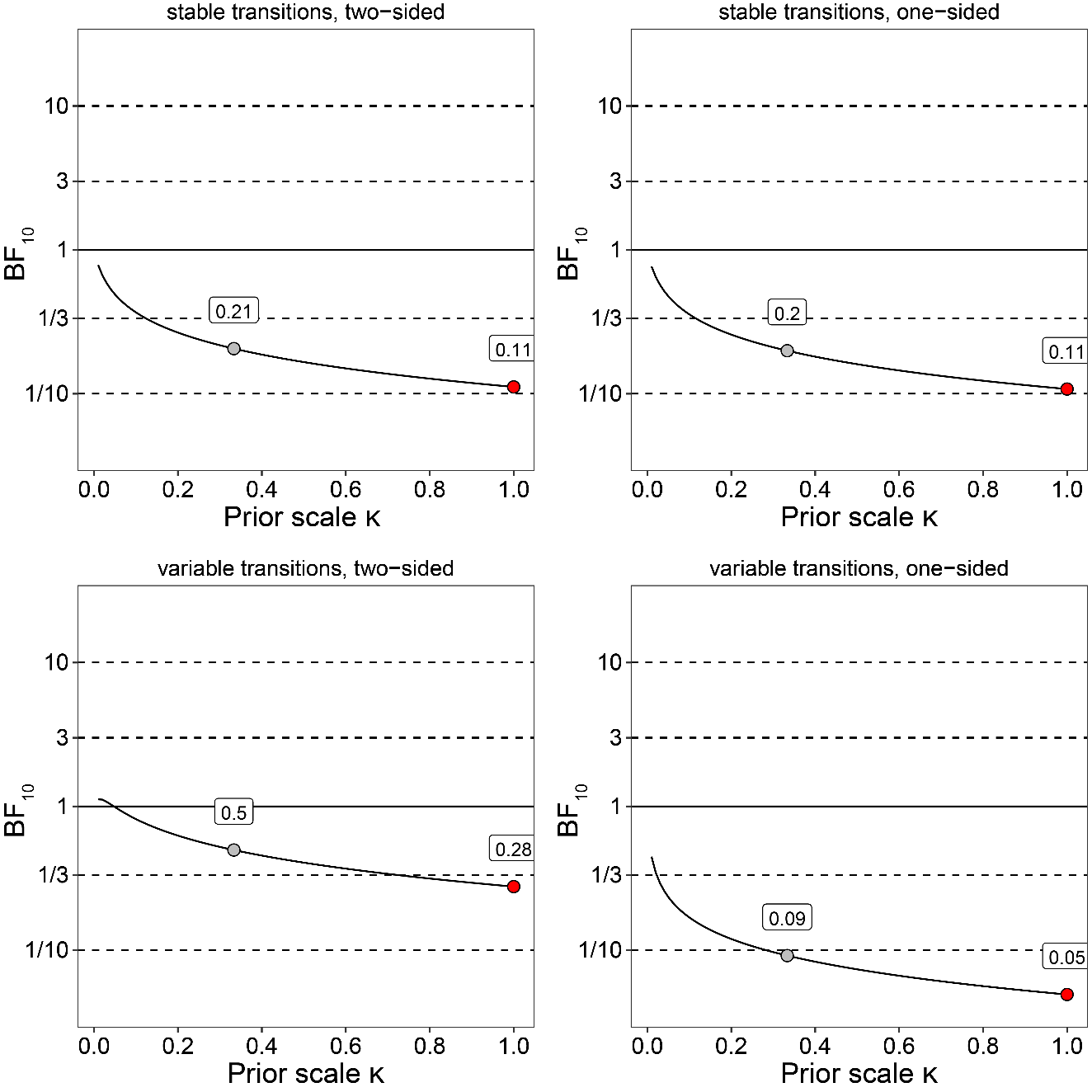** |  |
| **Figure S3**  *Bayes Factor robustness checks for the analyses of correlations between NFC and metacontrol in Study 2. The two-sided test compares evidence for a non-zero correlation and for no correlation; the one-sided test compares evidence for a negative correlation and for no correlation. Plots show Bayes Factors for different values of the scaling parameter κ (ranging between 0.01 and 1). For κ = 1, all population correlations between -1 and 1 are equally likely a priori and with smaller values of κ, correlations near zero become more likely a priori than correlations close to -1 or 1. Highlighted are the scaling value used in this study (κ = 1/3, red) and the scaling value used in Study 1 (κ = 1, gray)* | |
| **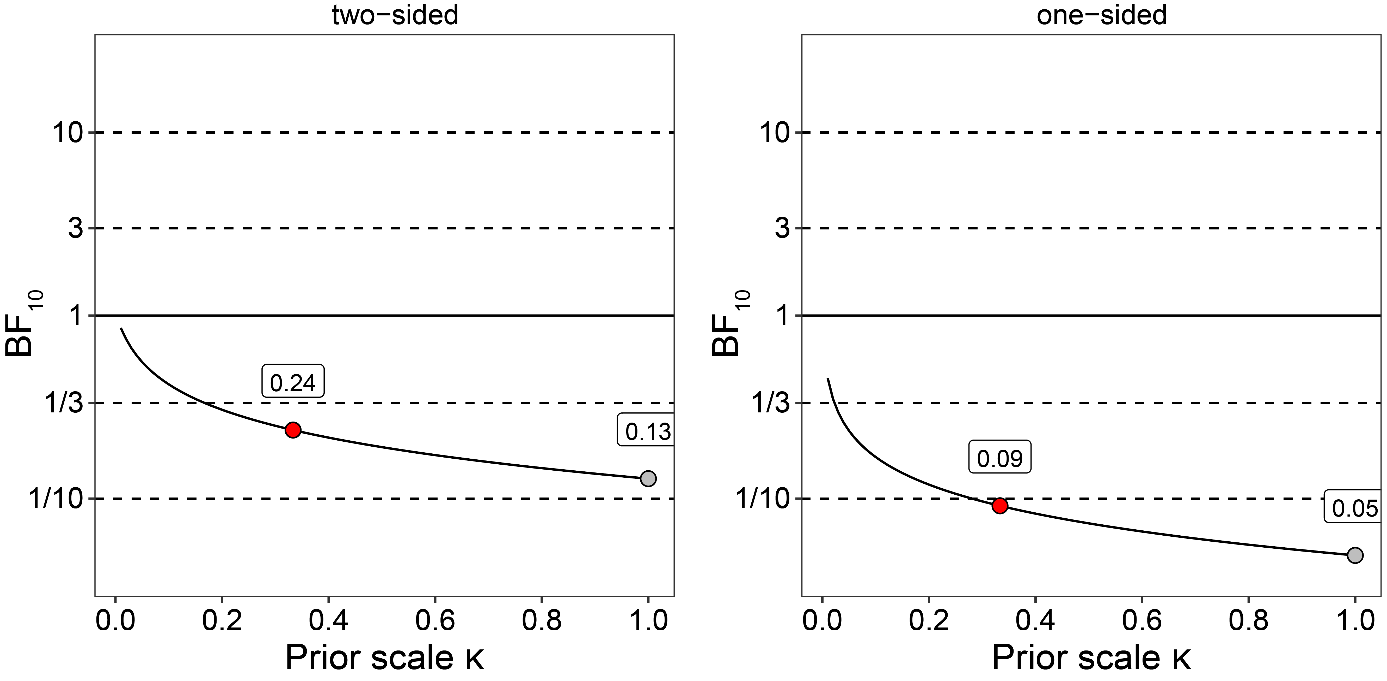** | |
